# The near-global ocean mesoscale eddy atmospheric-oceanic-biological interaction observational dataset

**DOI:** 10.1038/s41597-022-01550-9

**Published:** 2022-07-22

**Authors:** Changming Dong, Lingxiao Liu, Francesco Nencioli, Brandon J. Bethel, Yu Liu, Guangjun Xu, Jing Ma, Jinlin Ji, Wenjin Sun, Haixia Shan, Xiayan Lin, Bin Zou

**Affiliations:** 1 Ocean Modeling and Observation Laboratory, School of Marine Sciences, Nanjing University of Information Science and Technology, Nanjing, China; 2 Southern Laboratory of Ocean Science and Engineering (Guangdong Zhuhai), Zhuhai, China; 3 Collecte Localisation Satellites, Toulouse, France; 4 Marine Science and Technology College, Zhejiang Ocean University, Zhoushan, China; 5 College of Electronic and Information Engineering, Guangdong Ocean University, Zhanjiang, China; 6 National Satellite Ocean Application Service, Beijing, China

**Keywords:** Physical oceanography, Marine biology, Atmospheric dynamics

## Abstract

Amongst the variety of oceanic processes running the gamut of spatiotemporal scales, mesoscale eddies are the most common and often have region-specific characteristics. The large kinetic energy inherent to eddies themselves is a strong modulator of the global climate, ocean circulation, productivity, and freshwater transport. This study uses multi-source satellite remote sensing observation data to construct a multi-parameter eddy dataset for the 1993–2019 period, which differs significantly from a few of previous published eddy datasets that include only basic sea surface eddy physical features. Eddies within the dataset have life cycles of greater than four weeks, and their corresponding sea surface chlorophyll, sea surface temperature, and wind fields are provided. Atmospheric and oceanic variables are used to present a comprehensive picture of a given mesoscale eddy’s impact on the local physical, but also biological environment. The dataset would find immense value in research on mesoscale eddies, their impact on the atmosphere, and related biological processes.

## Abstract (summary)

**Table:** 

Measurement(s)	mesoscale eddy • chlorophyll • thermohaline profile
Technology Type(s)	remote sensing • argo float

## Background & Summary

Mesoscale eddies are ubiquitous in the global ocean and have important effects on climate, ocean circulation, and biological productivity. Since their initial discovery, eddies have changed scientific understanding of ocean circulation as they exist not only in the inner ocean but are also prevalent throughout marginal seas. A variety of methods for detecting eddies are used and include the Okubo-Weiss (O-W) algorithm^1–3^, 2D wavelets^4^, winding angles^5^, local extremes^6^, vector geometry^7^, Lagrangian Coherent Structures (LCSs)^8,9^, trajectory characterization method^10^ and most recently, artificial intelligence^11^, with research examples available throughout the global ocean^12–25^. Presently, several eddy datasets have been built and publicly distributed. These include the Chelton^3,6^, Mason^26^, Loop Eddy^27^, Three-Dimensional Eddy^28,29^, and the Faghmous^30^ datasets, amongst others. With the increasing prevalence and diversity of observational data, research into mesoscale eddies and their associated marine biogeochemistry processes has accelerated faster than previously imagined. The research team behind the present study has been an active participant in the study of eddies, demonstrated by a series of achievements.

Dong *et al*.^31^ used optical buoy data to identify a cold-core cyclone eddy near Lanai Island in the Hawaiian Archipelago, and through the combination of numerical simulations, and satellite-observed chlorophyll and sea surface temperature (SST) data, discovered a new type of eddy. Using oceanic velocity vector fields, a novel eddy detection algorithm was devised before being improved by Nencioli *et al*.^7^ that then applied it to high-resolution model data and altimeter observations in the Southern California Gulf. Dong *et al*.^15^ developed a set of algorithms for automatically detecting and tracking mesoscale eddies using SST data based on the two-dimensional velocity field vector geometric feature recognition method, which was successfully applied to the Kuroshio Extension Region (KER). Ma *et al*.^32,33^ conducted research on the three-dimensional response characteristics of the atmosphere in the KER to ocean eddies based on satellite observations and reanalysis products. There it was demonstrated that the momentum vertical mixing mechanism is the dominant factor in the ocean eddy’s influence on the atmosphere. That study also found that the atmospheric response to the mesoscale eddies in the KER has significant seasonal differences, which are closely related to the stability of the background atmospheric boundary layer. Shan *et al*.^34^ used an idealized Weather Research and Forecasting (WRF) atmospheric model experiment to explore the effect of eddies on the atmosphere. The results show that as the background wind speed increases, latent and sensible heat fluxes caused by cyclonic eddies and anticyclonic eddies all increase. Additionally, the 10 m wind speed, precipitation and boundary layer height all decreased with the increase of background wind speed. Ji *et al*.^24^ used multi-source data to study four areas in the North Pacific with strong eddy activity (i.e., KER, Subtropical Countercurrent Area, California Coastal Current, Aleutian Islands) that are geographically separated by thousands of kilometers and do not significantly affect each other. The researchers also investigated regional eddy differences and seasonal variability, and eddy response on the atmosphere. Results showed that characteristic eddy parameters (eddy kinetic energy, SST anomalies), and the stability of the atmospheric background (potential temperature, turbulent kinetic energy flux) are the primary reasons for the regional and seasonal discrepancies in eddy influence on the atmospheric boundary layer of each region. Sun *et al*.^35–37^ used SST observations to find that there are a considerable number of cyclonic eddies in the South China Sea with warm centers or anticyclonic eddies with cold centers. Combined with model data analysis, they found that the intensity of such abnormal eddies is usually weaker than the normal eddy, in which the degree of change in seawater temperature and salinity induced by the abnormal eddy is only 10% of the normal eddy. Through individual case analysis, it is found that the intrusion of Kuroshio warm water is an important factor influencing the generation and disappearance of abnormal eddies. Xu *et al*.^38^ developed an automatic recognition algorithm for the chlorophyll ring structure in response to an observed chlorophyll ring-structure phenomenon. After eight years of eddy detection in the North Pacific, they found that only about 1% of the eddy edges have high chlorophyll zones. This phenomenon not only occurs at the edge of the anticyclonic eddy in previous studies, but also has the ring-shaped feature on the edge of the cyclonic eddy. Research suggests that symmetrical instability, the horizontal transport of chlorophyll in the surrounding water by the eddy, and vertical transport caused by the eddy-induced Ekman pumping at the edge of the eddy may be the underlying mechanism for the formation of the chlorophyll ring structure. Yang *et al*.^25^ also found this structure in Bay of Bengal eddies. At the same time, a quantitative analysis of the abnormal chlorophyll eddy found that the cyclone can increase the chlorophyll concentration of the surrounding sea surface by about 11%, while the anticyclone decreased the chlorophyll concentration. It was also identified that chlorophyll concentration changes rapidly in the mature and intensification phases of the eddy. Xia *et al*.^39^ identified coherent mesoscale eddies and estimated the coherent mass transport associated with them in the upper global ocean using a Lagrangian-averaged vorticity deviation (LAVD) method based on research into coherent Lagrangian vortices conducted by Haller *et al*.^40,41^.

In summary, it is important to study the interactions between eddies, the atmosphere, and marine biogeochemistry for climate change, ocean primary productivity, and global biological resource studies. Based on multi-source satellite remote sensing observation data, an automatic algorithm based on the velocity vector geometry of the oceanic flow field and a spatiotemporal matching method are used to detect and track eddies and couple an array of oceanic and atmospheric variables. Through this methodology, the near-global ocean mesoscale eddy atmospheric-oceanic-biological interaction observational dataset (GOMEAD) can be built. Distinct from other publicly available eddy databases, this database not only contains fundamental eddy characteristics data, but also includes eddy-related ocean-atmosphere-biology information. This information includes sea surface chlorophyll (SSC), SST, eddy thermohaline profiles, sea surface wind fields, precipitation rate, in addition to water vapor and cloud liquid water content. This database contains a considerable amount of data that can be used either directly in relevant research or can be complemented with other satellite or *in situ* observational data so that a deeper understanding of eddy basic physical characteristics and Earth’s biosphere can be achieved. Additionally, as the database can be used to advance the study of eddies and related phenomena, it is potentially of great significance to the study of global ocean mesoscale processes.

## Methods

### Eddy detection and tracking

The data used for automatic eddy detection in this study is from “all-satellite” daily Delayed Time (DT) DUACS (Data Unification and Altimeter Combination System) 2018 version (https://cds.climate.copernicus.eu/), the selected time span is from January 1, 1993 to December 31, 2019. Automatic eddy detection is based on daily datasets with a spatial resolution of 1/4°. The dataset includes the characteristic information of sea level anomalies, absolute geostrophic velocity, and geostrophic velocity anomalies. The eddy detection method used in this study is based on an automatic eddy recognition algorithm based on geometric characteristics of flow field proposed by Nencioli *et al*.^7^, from which a detailed description can also be found. The following is a brief description of the detection method.

First, the center of the eddy is determined by the geometry of the velocity vector through four constraints:

1. Along the east-west direction of the eddy center, the signs of the velocity component ${v}^{{\prime} }$ on both sides of the center are opposite, and the magnitude increases gradually with the distance from the center;
2. Along the south-north direction of the eddy center, the signs of the velocity component ${u}^{{\prime} }$ on both sides of the center are opposite, and the magnitude increases gradually with the distance from the center, ${u}^{{\prime} }$ changes in the same direction as ${v}^{{\prime} }$;
3. Velocity magnitude has a local minimum at the eddy center;
4. Around the eddy center, the directions of the velocity vectors have to change with a constant sense of rotation and the directions of two neighboring velocity vectors have to lay within the same or two adjacent quadrants (the four quadrants are defined by the east-west and north-south axes: the first quadrant encompasses all the directions from east to north, the second quadrant encompasses the directions from north to west, the third quadrant encompasses the directions from west to south, and the fourth quadrant encompasses the directions from south to east). The meridional and zonal components are calculated by using AVISO sea level anomaly as follows (Eqs. (1) and (2)):

1 $${u}^{{\prime} }=-\frac{g}{f}\left(\frac{\partial {h}^{{\prime} }}{\partial y}\right)$$

2 $${v}^{{\prime} }=\frac{g}{f}\left(\frac{\partial {h}^{{\prime} }}{\partial x}\right)$$

where ${h}^{{\prime} }$ represents sea level anomaly, f is Coriolis parameter, and g is gravitational acceleration):

The grid point satisfying the above four constraints is automatically judged as the center of eddy. The constraints require two parameters to be specified in the vector geometry-based eddy detection algorithm: one for the first, second, and fourth constraints and one for the third one. The first parameter, a, defines how many grid points away the increases in magnitude of y along the E-W axes and u along the N-S axes are checked. It also defines the curve around the eddy center along which the change in direction of the velocity vectors is inspected. The second parameter, b, defines the dimension (in grid points) of the area used to define the local minimum of velocity. For the flow field of altimeter geostrophic velocity anomalies used in this paper, the optimal value is tested as *a* = 4, *b* = 3. In order to further improve the performance of the algorithm, the AVISO velocity field is linearly interpolated into a 1/6° × 1/6° grid before the application of the detection method, so that the eddy current velocity field is extended to more grid points.

After the center of the eddy is determined, the closed streamline of the periphery of the eddy is detected. In this algorithm, the boundary of eddy is defined by isoline of the stream function. As the eddy velocity field is weakly divergent, the velocity vector is tangent to the isoline of the flow function. The tangential velocity of the flow increases along the normal direction. The eddy boundary is defined as the outermost closed isoline around the center. Finally, the motion trajectory of the eddy is calculated, and the splitting and merging of the eddy is considered. After the eddy center is identified from the sea level anomaly data, the track of the eddy is determined by comparing the center position in over time. The center of eddy at time t + 1 has the same polarity as the grid point range of N × N at time t. In this way, the eddy center at time t can be associated with the eddy center at time t + 1. If no connected center is found at time t + 1, a larger area (N + N/2) × (N + N/2) is searched at time t + 2. Here, N is distance in km to define the area used to derive eddy tracks, set according to the grid number. In particular, to avoid splitting a continuous track into multiple tracks, eddies cannot travel outside the searching area from one time step to the successive step. In case more than one eddy of the same type is found within the searching area, either at t + 1 or t + 2, the eddy track is updated with the closest center to the eddy at t. When no centers are detected within the searching area at t + 2, the eddy is considered as dissipated and the track for that specific eddy is closed. On the other hand, eddy centers at t + 2 that were not connected to any eddy center at t are considered newly formed eddies, and their tracks will be updated starting from time step t + 3. The size of eddy search range needs to be carefully quantified in the next step to avoid the disassembly of eddy track (the continuous eddy track is divided into multiple tracks).

### Pairing eddies with oceanic, atmospheric, and biological elements

Detected eddies are automatically paired with their respective oceanic, atmospheric, and biological components to form the global ocean mesoscale eddy ocean-atmosphere-biology coupled observation data database. Database data are collated from several sources. Multi-satellite fusion chlorophyll products with spatial resolution of 4 km × 4 km and temporal resolution of eight days provided by Globcolour database in HERMES (http://hermes.acri.fr), used to study changes in the distribution of chlorophyll on the sea surface around the eddies, the selected time span is from August 29, 1997 to December 31, 2019. Thermohaline profile dataset (ftp://ftp.ifremer.fr/ifremer/argo/geo/) with 1 day temporal resolution from Argo international project home page (http://www.argo.nrt/) are used to study the vertical influence of eddies on the surrounding water body and the vertical characteristics of eddies. The selected time span is from September 7, 1995 to December 31, 2019. SST data (https://www.ncei.noaa.gov/thredds/catalog/OisstBase/NetCDF/V2.0/AVHRR/catalog.html) with spatial resolution of 25 km × 25 km and temporal resolution of one day obtained from National Aeronautics and Space Administration (NASA), as an auxiliary material for studying abnormal eddies. The selected time span is from January 1, 1993 to December 31, 2019. Gridded daily wind speed, cloud liquid water content, water vapor content, and rainfall rate with a horizontal resolution of 0.25° × 0.25° provided by AMSR-E and AMSR-II level 3 products (https://www.remss.com/missions/amsr/), for studying the interaction of eddies with the atmosphere, the selected time span is from June 1, 2002 to December 31, 2019.

**Fig. 1:**
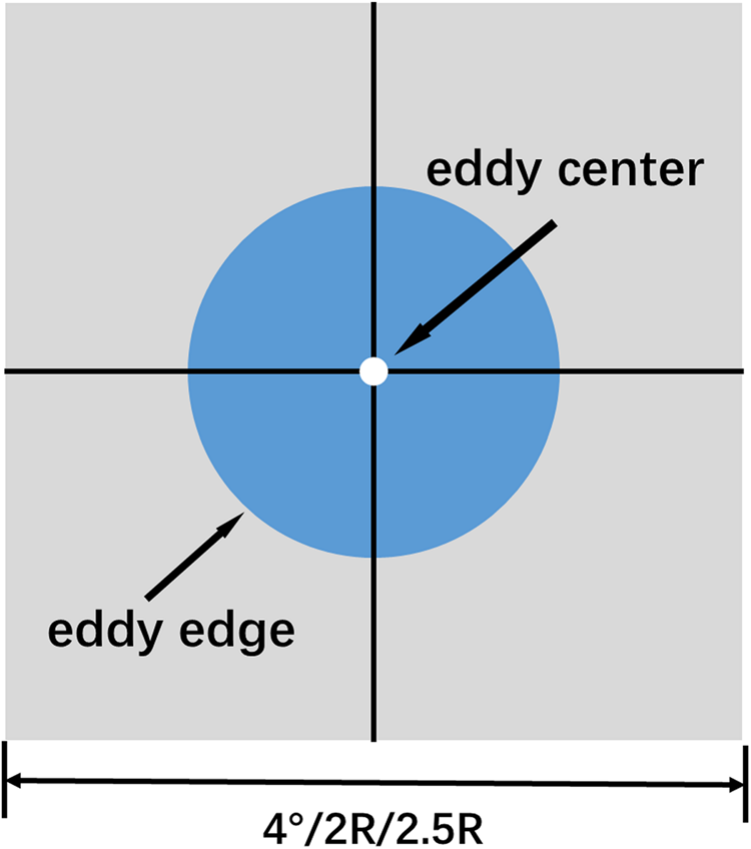
Sketch of an eddy matched with each element. The white dots represent the eddy center, the blue filled circles represent the eddy, and the gray rectangular area is the box frame area extended by a specified distance (4°/2 R/2.5 R) from the eddy center as the origin.

In this study, oceanic, atmospheric and biological elements associated with eddies are extracted by using a spatial-temporal matching method that comes in two steps. Firstly, the region has its origin at the center of the eddy and takes 2.5/2 times the eddy radius or 4° × 4° around the eddy center in the middle and high latitudes or higher as the spatial scale limit to expand in both the meridional and latitudinal directions, as the matching area of the background field (the eddy radius was defined as the average of the distance from the eddy center to the eddy boundary). Secondly, based on the temporal and spatial overlap of data, the background elements’ fields of an individual eddy are extracted and integrated. For the chlorophyll data, there are a lot of missing values in the daily data, which cannot be applied well. Therefore, the 8-day average data is selected. Due to the difference in time resolution between water color data and eddy information, this study assumes that if an eddy exists for a time corresponding to the initial range of chlorophyll data, it is used as the background field of the eddy. A schematic diagram of the matching method is shown in Fig. 1.

## Data Records

The near-global ocean mesoscale eddy atmospheric-oceanic-biological interaction observational dataset^42^ (GOMEAD, 10.11922/sciencedb.01190) can be accessed through 10.11922/sciencedb.01190. The dataset is 1.48 TB in total (only refers to data files in netCDF format), consisting of several subfolders representing four different regions, such as North Pacific (NP: 5°–65°N, 100°–260°E), North Atlantic (NA: 5°–65 °N, 260°–360°E), North Indian Ocean (NI: 5°–30°N, 45°–100°E) and Southern Hemisphere Ocean (SHO: 5°–65°S, 0°–360°E). Each subfolder includes eddy tracking files (*eddy_tracks.nc*) which are named by eddy identification numbers (*ID.nc*). These eddies are those with lifetimes of greater than four weeks. Both data are based on Lagrangian statistics, i.e., based on eddy lifetime storage. The first set of data contains all trajectories and their identifiers, and the second contains paired data for each eddy in the entire eddy trajectory. Each file consists of basic eddy features (polarity, central longitude and latitude, size, lifetime and geostrophic current anomaly, etc.) which are paired with matched information such as sea surface chlorophyll a concentration, Argo temperature and salinity profiles, sea surface temperature and atmospheric elements (precipitation rate, 10 m wind, cloud liquid water content, moisture content, etc.). Excluding the eddy tracking file itself, the size of each MATLAB struct file will not exceed 100 MB. File specific fields are as follows:

### Eddy Tracking File (*eddy_track.nc*)

1. ID: Identification number of the eddy. All observations within the same trajectory have the same ID.
2. time: Date of the detected eddy (serial date numbers, preset date (January 0, 0000) in the proleptic ISO calendar).
3. eddy_type: Polarity of the eddy. 1 represents cyclonic, −1 represents anticyclonic.
4. center_latitude: Latitudes of the eddy center.
5. center_longitude: Longitudes of the eddy center.
6. shapes_latitude: Latitudes of the eddy boundary.
7. shapes_longitude: Longitudes of the eddy boundary.
8. radius: Radius of the eddy (m).
9. tracks: Observation sequence number, days from eddy first detection.

**Eddy information file (*ID.nc*)** (Note: If there is no matching element, it will be displayed as NAN or -2147483648)

1. ID: Identification number of the eddy.
2. time: Date of the detected eddy (serial date numbers, preset date (January 0, 0000) in the proleptic ISO calendar).
3. eddy_type: Polarity of the eddy. 1 represents cyclonic, −1 represents anticyclonic.
4. radius: Radius of the eddy (m).
5. shapes: Longitudes and latitudes of the eddy boundary.
6. center: Longitudes and latitudes of the eddy center.
7. argo: Longitudes and latitudes of Argo matched within 2 times radius of the eddy.
8. argo_dimension: Argo vertical profile data dimensions (levels) matched within 2 times radius of eddy.
9. argo_tracks: Argo corresponds to the observation sequence number, days from eddy first detection.
10. argo_type: Type of Argo matched within 2 times radius of the eddy (R: real-time; D: delayed; A: real-time adjustment).
11. argo_press: Potential density profile (kg/m^3^) obtained by the Argo matched within 2 times radius of the eddy.
12. argo_sals: Salinity profile (psu) obtained by the Argo matched within 2 times radius of the eddy.
13. argo_temps: Potential temperature profile (degree C) obtained by the Argo matched within 2 times radius of the eddy.
14. sst_lon: Longitudes of sea surface temperature matched within 2.5 times radius of the eddy.
15. sst_lat: Latitudes of sea surface temperature matched within 2.5 times radius of the eddy.
16. sst_dimension: Sea surface temperature data dimensions (sst_lon, sst_lat, sst_lon × sst_lat) matched within 2.5 times radius of eddy.
17. sst: Sea surface temperature (degree C) matched within 2.5 times radius of the eddy.
18. uv_lat: Latitudes of geostrophic current anomaly matched within 2.5 times radius of the eddy.
19. uv_lon: Longitudes of geostrophic current anomaly matched within 2.5 times radius of the eddy.
20. ssuv_dimension: Geostrophic current anomaly data dimensions (uv_lon, uv_lat, uv_lon × uv_lat) matched within 2.5 times radius of eddy.
21. ssu: Meridional component of geostrophic current anomaly (m/s) matched within 2.5 times radius of the eddy.
22. ssv: Zonal component of geostrophic velocity anomaly (m/s) matched within 2.5 times radius of eddy.
23. amsr_lat: Latitudes of atmospheric background field matched within the range of 4° × 4° outward from the eddy center.
24. amsr_lon: Longitudes of atmospheric background field matched within the range of 4° × 4° outward from the eddy center.
25. amsr_dimension: Atmospheric background field data dimensions (amsr _lon, amsr _lat, amsr _lon × amsr _lat) matched within the range of 4° × 4° outward from the eddy center.
26. wspdLF: Low-frequency 10-meter wind speed (m/s) matched within the range of 4° × 4° outward from the eddy center (10.7 GHz and above).
27. wspdMF: Intermediate-frequency 10-meter wind speed (m/s) matched within the range of 4° × 4° outward from the eddy center (18.7 GHz and above).
28. vapor: Water vapor content (mm) matched within the range of 4° × 4° outwards from the eddy center.
29. cloud: Cloud liquid water content (mm) matched within the range of 4° × 4° outwards from the eddy center.
30. rain: Precipitation rate (mm/h) matched within the range of 4° × 4° outwards from the eddy center.
31. chl_lat: Latitudes of SSC matched within 2.5 times radius of the eddy.
32. chl_lon: Longitudes of SSC matched within 2.5 times radius of the eddy.
33. chl_dimension: SSC data dimensions (chl_lon, chl_lat, chl_lon × chl_lat) matched within 2.5 times radius of eddy.
34. chl: SSC (mg/m^3^) matched within 2.5 times radius of the eddy.

## Technical Validation

### Expert detection system verification

Within the dataset, as oceanic eddies are derived physical phenomena and not directly observed in the real ocean, true values are necessarily unknown. Indeed, the velocity vector geometry scheme includes a variety of elastic detection parameters, sensitivity analyses of these parameters are a required and not merely optional step. Assumed to be detected eddies are compared at random with a manually detected eddy field and an effective evaluation is determined by eddy detection success and false alarm rates^43^. These are determined as follows (Eqs. (3) and (4)):

3 $$SDR=\frac{{N}_{c}}{{N}_{te}}\times 100$$

4 $$EDR=\frac{{N}_{oa}}{{N}_{te}}\times 100$$

where SDR is the success of detection rate, EDR is the excess of detection rate. ${{\rm{N}}}_{{\rm{te}}}$ is the number of eddies identified by experts, i.e., the number of true eddies, ${{\rm{N}}}_{{\rm{c}}}$ is the number of eddies identified by the vector geometric algorithm that agree with those identified by the expert, which is the real eddy center detected by the vector geometric identification algorithm, and ${{\rm{N}}}_{{\rm{oa}}}$ is the difference between all eddies identified by several identification algorithms and ${{\rm{N}}}_{{\rm{c}}}$, i.e., the number of false eddies. The algorithm evaluation has been compared and verified by different parameter combinations in the Southern California Bay^7,16^, the Subtropical Countercurrent zone^18^, the South China Sea^21^, the Bay of Bengal^25^, the Gulf Stream, and Tasman Sea. From these analyses, the best value combination of the parameters for global eddy detection can be comprehensively selected, taking into account the universality of most ocean areas around the world (a = 4, b = 3). Check and evaluation of the eddy algorithm for the above six sea areas under the optimal parameter combination is shown in Table 1 (due to the large number of parameter combinations error test results, the manuscript does not show them one by one, but only shows the error test results of the optimal combination). The results of multiple evaluations in different regions show that the geometry scheme has a lower over-detection rate (${{\rm{EDR}}}_{{\rm{mean}}}\approx 3.9{\rm{ \% }}$) and a higher detection success rate (${{\rm{SDR}}}_{{\rm{mean}}}\approx {\rm{94}}.8{\rm{ \% }}$), which can accurately capture the eddy structures, and thus can be used reliably for eddy-related studies.

**Table 1:** Randomized 10-day eddy validation results for six regions under a = 4, b = 3 parameter combination.

Region	Rate (%)	Day 1	Day 2	Day 3	Day 4	Day 5	Day 6	Day 7	Day 8	Day 9	Day 10
Southern California Bay	EDR	0.0	10.0	0.0	10.0	0.0	5.6	0.0	0.0	3.8	0.0
	SDR	94.7	85.0	93.3	95.0	93.7	94.4	93.8	100.0	88.5	91.3
South China Sea	EDR	18.8	14.7	10.8	7.4	6.3	7.5	9.8	11.9	15.8	11.4
	SDR	100.0	94.1	89.2	96.3	96.9	90.0	97.6	100.0	97.4	97.2
Bay of Bengal	EDR	0.0	0.0	3.8	3.1	0.0	4.2	2.9	0.0	5.9	0.0
	SDR	96.7	95.7	88.5	96.7	97.3	87.5	97.1	94.2	100.0	94.3
Subtropical Countercurrent Zone	EDR	0.0	2.9	1.4	3.2	0.0	10.0	4.8	5.4	0.0	1.5
	SDR	97.0	95.6	95.7	93.5	95.2	90.1	96.8	94.6	91.5	84.4
Gulf Stream	EDR	6.5	1.5	0.0	2.5	2.9	0.0	5.0	2.5	0.0	0.0
	SDR	93.5	90.3	96.9	97.4	97.1	97.8	95.0	97.5	97.1	92.9
Tasman Sea	EDR	2.0	2.2	0.0	2.1	5.1	2.2	0.0	4.0	1.9	0.0
	SDR	92.0	95.6	95.5	93.8	94.9	95.5	98.0	100.0	100.0	98.0

### Eddy algorithm and data application

Both the velocity vector geometry scheme using altimeter observations and the present ocean eddy dataset derived from this scheme has been extensively utilized in previous studies. For example, Dong *et al*.^17^ first applied the scheme to estimate average eddy heat and salt transport from vertical profiles from co-located Argo floats. Eddies were initially identified from altimeter observations of sea surface height (and calculated anomalies). It was identified that temperature and salinity (T/S) anomalies inside individual eddies tend to move with eddies because of advective trapping of interior water parcels. The estimated meridional heat transport by eddy movement is similar in magnitude and spatial structure to previously published eddy covariance estimates from models. Eddy heat and salt transports both are a sizeable fraction of their respective total transports. The primary heat transport occurs in the tropical, subtropical and subpolar zones. Liu *et al*.^18^ conducted a comprehensive analysis of the basic physical characteristics and evolution of eddies in the subtropical countercurrent zone of the North Pacific based on the velocity vector geometry scheme and showed that baroclinic instability and lee side wind stress curl is responsible for the eddy generation. Qin *et al*.^20^ found that the oceanic eddies in the East China Sea is mainly controlled by Kuroshio transport. Lin *et al*.^21,22^ applied the eddy detection scheme to an eddy-resolving numerical solution to build a three-dimensional eddy dataset in the South China Sea. Three different types of eddies were identified: bowl-shaped with the largest size at the surface, lens-shaped with the largest size in the middle and cone-shaped with the largest size at the bottom. Ji *et al*.^23,44^ analyzed the characteristics of the oceanic eddies over the Kuroshio Extension region, and found that more anticyclonic (cyclonic) eddies with lifetimes longer than 50 weeks are located to the north (south) of 35°N, while more cyclonic (anticyclonic) eddies with lifetimes shorter than 20 weeks occur north (south) of 35°N. This asymmetric distribution of eddies suggests two eddy generation mechanisms: (1) the development of meanders in the Kuroshio path led to the shedding of eddies with longer lifetimes, and (2) horizontal shear instability (barotropic instability) which led to eddies with shorter lifetimes. Maúre *et al*.^45^ investigated the effect of mesoscale oceanic eddies on phytoplankton blooming events of the Sea of Japan Yamato Basin and found that there was difference in blooming times and initiation mechanisms between cyclones and anticyclones. Rena *et al*.^46^ tracked oceanic eddies in the eastern tropical South Pacific and found that they would mix horizontally with the surrounding water masses. More oxygen-enriched water, warm water, and salt water were entrained in the upper part of anticyclones than in cyclones. Müller *et al*.^47^ built upon prior work to construct a thermohaline and oceanic eddy dataset to study heat and freshwater transports associated with eddies. It was demonstrated that approximately 25% of the absolute temperature flux by eddies across 47°N stems from strong eddies. Androulidakis *et al*.^48^ found that the upwelling accompanied by the oceanic eddies in the Gulf of Mexico can make the Gulf Stream more active. Hu *et al*.^49^ investigated the forcing effect of the North Pacific oceanic eddies on the mid-latitude atmosphere, finding both local and remote effects. In addition, various eddy detection methods have also been compared and verified. Jiang *et al*.^50^ compared the O-W method and the velocity vector geometry scheme and showed that both methods can adequately identify mesoscale eddies and capture their basic characteristics, but the geometry scheme can detect more eddies. Wekerle *et al*.^51^ applied both the velocity vector geometry scheme and the O-W method to the Regional Ocean Model System (ROMS) and Finite-Element Sea-Ice Ocean Model (FESOM) models. It is found that most eddies were detected, and the geometry scheme avoided an over-detection by the O-W method. Xu *et al*.^52^ used three artificial intelligence algorithms based on deep learning semantic segmentation to identify mesoscale eddies. Eddies detected by these methods are compared with each other and with the results from the traditional method, which illustrate the accuracy and practicability of the three methods. In summary, the automatic detection and tracking of mesoscale oceanic eddies based on the velocity vector geometry scheme has high application and research value.

### The statistic and analysis of eddy characteristics

**Fig. 2:**
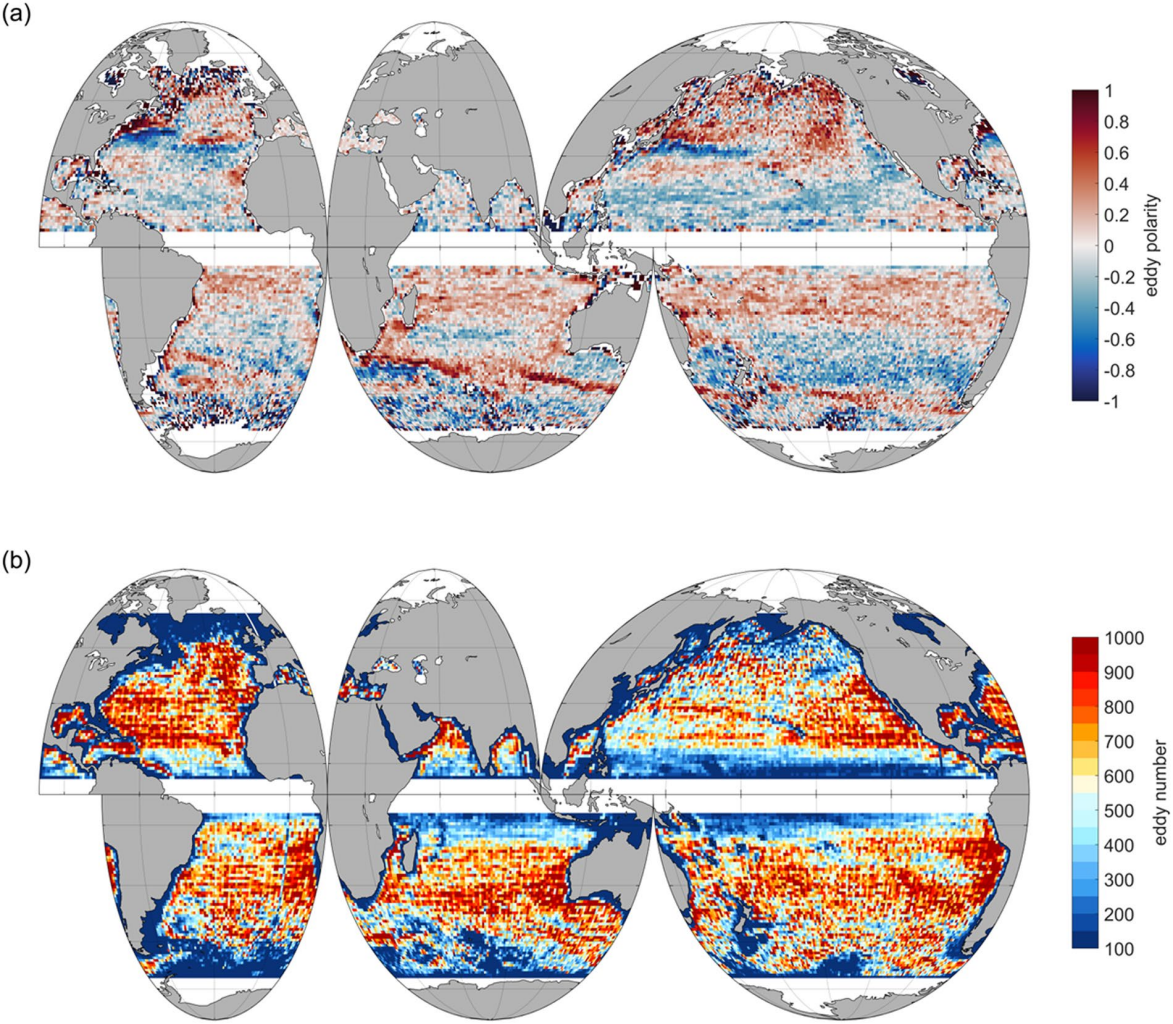
The spatial distributions of eddy polarity and number in a 1° × 1° grid where (**a**) is average eddy polarity, defined as (${\rm{Na}}-{\rm{Nc}}$)/(${\rm{Na}}+{\rm{Nc}}$), where ${\rm{Na}}$ is the number of anticyclonic eddies and ${\rm{Nc}}$ is the number of cyclonic eddies, and (**b**) is average eddy number.

Considering the influence of the altimeter data, and based on the Lagrangian counting method, a total of 269,716 eddies with a life cycle longer than 4 weeks were identified from January 1, 1993 to December 31, 2019. There were a total of 134,886 anticyclonic and 134,830 cyclonic eddies. By contrast, when using the Eulerian counting method, a total of 15,051,799 eddies were detected of which 7,498,407 are cyclonic eddies and 7,503,392 are anticyclonic eddies. Here, anticyclonic eddies are more numerous than cyclonic eddies by 0.60%. Moreover, the spatial distribution of eddies within the global ocean is slightly different. For example, there are notably more cyclonic eddies in the Northern Indian and Atlantic Oceans than there are anticyclonic eddies. This phenomenon is reversed in the North Pacific and Southern Hemisphere Ocean. When the globe is divided into 1° × 1° grid points and the number of eddies is filtered through each moment in time using the Eulerian counting method, it can be observed in Fig. 2 that the spatial distribution of the two eddy types is roughly similar. In areas with strong currents such as the Kuroshio, Gulf Stream, and Antarctic Circumpolar Current etc., the southern Indian Ocean and eastern Atlantic Ocean possess more eddies, consistent with previous studies^3,6^. However, the difference is that there are also many eddies near the California and North Pacific Currents, the Peruvian Basin, and the Chilean Trench. The distribution of cyclonic eddies is denser than that of anticyclonic eddies and this is especially true in the Atlantic Ocean. There, cyclonic eddies are primarily concentrated in the midlatitudes, but anticyclonic eddies are found closer to shorelines. Both eddy polarities are distributed in bands from the Subtropical Countercurrent to the poles in the northwestern Pacific Ocean. Using 35°N as the dividing line, cyclonic eddies are more numerous than anticyclonic eddies south of 35°N. The opposite is true in the north, and is also the case in the North Atlantic Ocean. Comparing both cyclonic and anticyclonic eddies identified and tracked in this study with the results of Griffa *et al*.^27^ that used data from Argo drifters, it is found that within the northern hemisphere, two latitudinal bands exist. The first is the belt of anticyclonic eddies from 30°–40°N, and another is the cyclonic eddy belt from 10°–20°N. The southern Indian Ocean also maintains a distribution similar to that of the northern hemisphere. However, differing from the South Atlantic Ocean, the South Pacific Ocean has most cyclonic eddies concentrated from 25°–40°S, and anticyclonic eddies located from 15°–30°S.

**Fig. 3:**
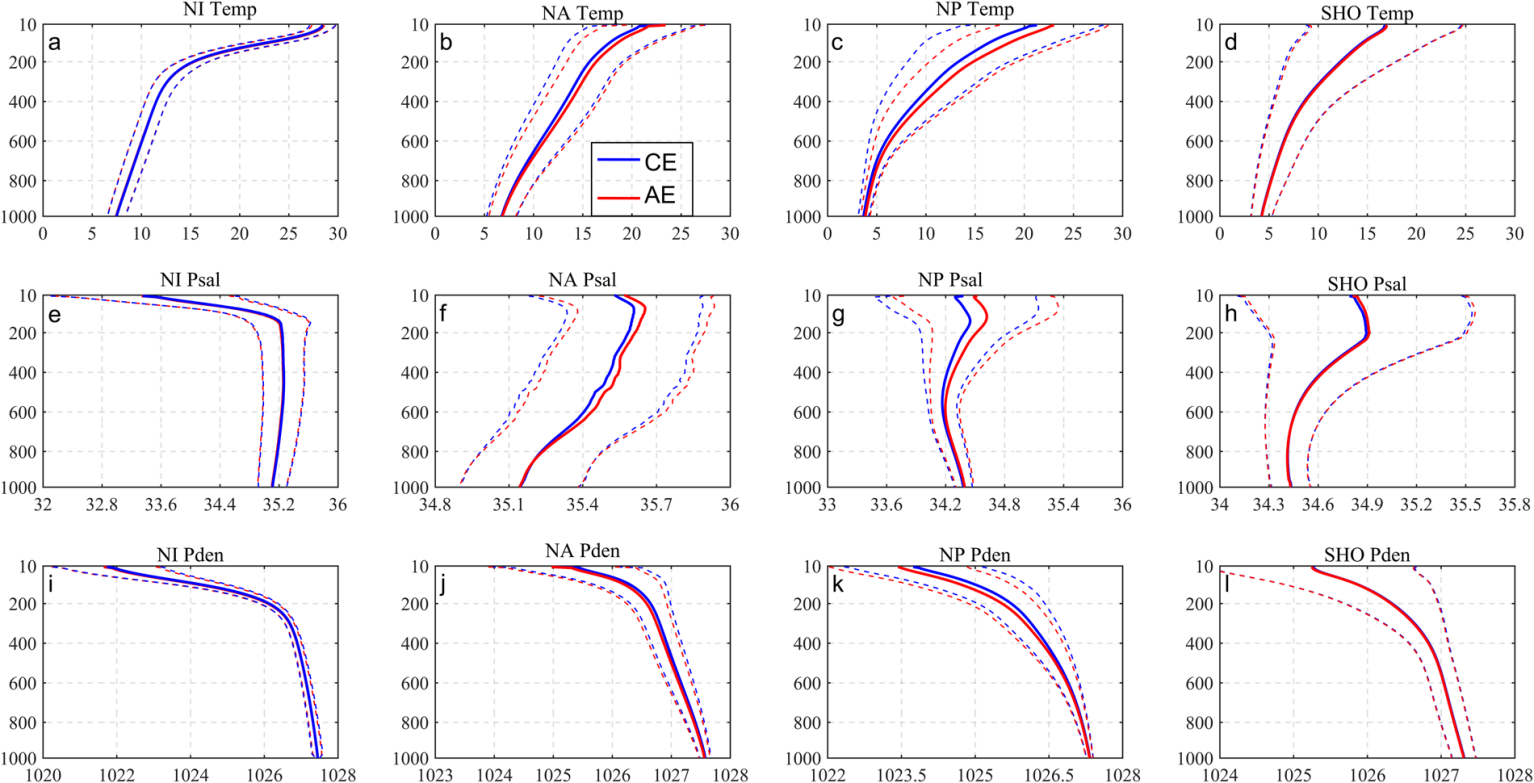
Comprehensive average vertical distribution of eddy temperature (top), salt (middle), and density (bottom). From left to right are the northern Indian (**a**), (**e**), (**i**), North Atlantic (**b**), (**f**), (**j**), North Pacific (**c**), (**g**), (**k**) and the Southern Hemisphere Ocean (**d**), (**h**), (**l**). Blue represents cyclonic eddies, red represents anticyclonic eddies, and the dotted line is the standard error of the mean.

To explore the vertical characteristics of eddies at the different depth of ocean, we use Argo data to study the influence of anticyclonic and cyclonic eddies on the vertical structure of the upper ocean temperature and salt, respectively. There were 1,550,287 Argo T/S profiles in the global ocean during the period of 1995 to 2019. The limitation which used to define the Argo float trapped by eddies is that the distance of Argo float to the eddy center should be less than twice the radius of the eddy. Once this criterion is satisfied, an eddy T/S profiles database can be constructed. During the 27 years, a total of 814,531 (834,757) Argo profiles have been captured by cyclonic (anticyclonic) eddies. Moreover, there are 352,844 eddies (177,084 anticyclonic eddies /175,760 cyclonic eddies) that can be paired with at least one Argo profile. To eliminate data with unreasonable error, quality control is performed using triple standard deviation on Argo data paired with eddies. T/S and density profiles in 10 meters are uniformly-spaced from 10 m to 1000 m in the vertical. Fig. 3 shows the T/S and density profiles vertical distributions in different regions. As the depth increases, the temperature gradually decreases, the salinity and density gradually increase. Within a depth range of 150–250 m, eddy internal temperatures and salinities are significantly changed, respectively. There are minor discrepancies between cyclonic and anticyclonic eddies. At depths of less than 400 m, temperature, salinity, and density profiles in either cyclonic or anticyclonic eddies, gradually converge with increasing depth. However, at the same time, the salinity section of the four seas shows different trends. The salinity of NI and NA continues to decrease. NP and SHO appear at 600–800 m to increase to increase. There are minor discrepancies between cyclonic and anticyclonic eddies. T/S profiles between the eddy types are also remarkably similar. An example is provided in Fig. 3. There, as compared with the North Atlantic, North Pacific, and Southern Hemisphere Ocean, eddies in North Indian Ocean are characterized by high temperatures, with low salinities and densities.

**Fig. 4:**
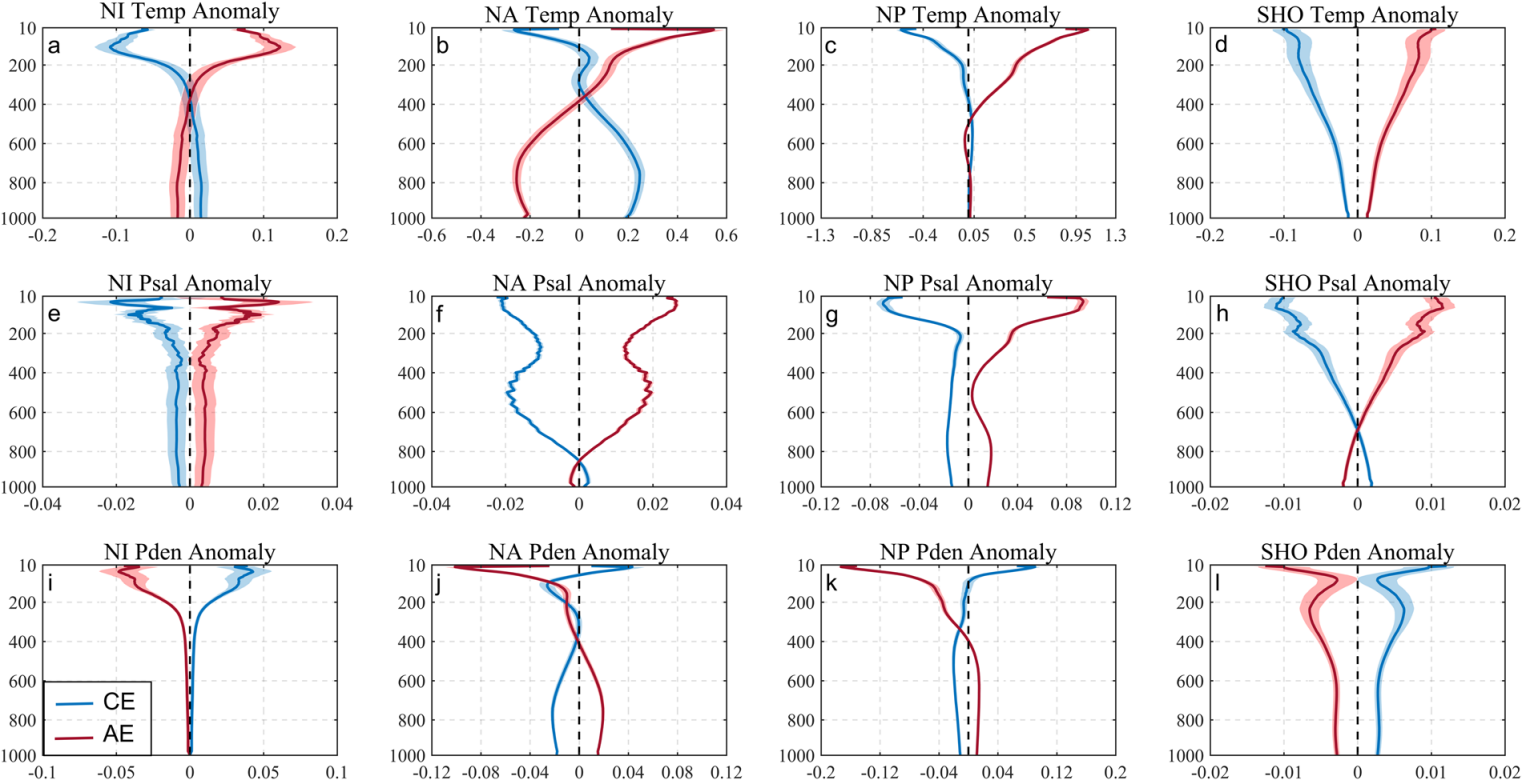
Comprehensive average vertical profiles of eddies temperature (top), salinity (middle) and density (bottom) anomalies. From left to right are the northern Indian (**a**), (**e**), (**i**), North Atlantic (**b**), (**f**), (**j**), North Pacific (**c**), (**g**), (**k**) and the Southern Hemisphere Ocean (**d**), (**h**), (**l**). Cyclonic eddies are represented by blue, anticyclonic eddies are represented by red, and shaded areas are the standard error of the mean.

Figure 4 shows T/S profile anomalies as induced by eddies. The anomalies here are the difference between the Argo vertical profiles captured by eddies in each sea area and the climatic profile data of the entire sea area. There, it can be observed that eddies can influence T/S profiles to a depth of 500–1000 m or more. Cyclonic (anticyclonic) eddies can cause negative (positive) anomalies in the T/S profile and positive (negative) density anomalies, particularly in the upper ocean. Vertical profiles of eddies in the North Indian Ocean, the North Pacific, and the Southern Hemisphere Ocean most in the lens-shaped. Within the North Indian and the North Pacific, as water depth increases, regardless of eddy polarity, temperature, salinity and density values first increase to extreme values at approximately a depth of 100–200 m, before decreasing, and the North Indian temperature slightly greater than the maximum depth salinity and density anomaly maximum depth, North Pacific is anomalous salinity maximum depth is larger. In contrast, although there is an anomalous local maximum value at about 200 m in the Southern Ocean, it is weaker than the near-surface. By contrast, inflection point depths range not from 150–250 m but is extended to the near surface and ~600 m in the North Atlantic Ocean, which is “gourd-shaped”. This may be related to the basin’s hydrodynamic environment. The magnitudes of North Atlantic and Pacific Ocean anticyclonic eddies are slightly greater than that of cyclonic eddies which may suggest that anticyclonic eddies may have a greater impact on the oceanic environment.

**Fig. 5:**
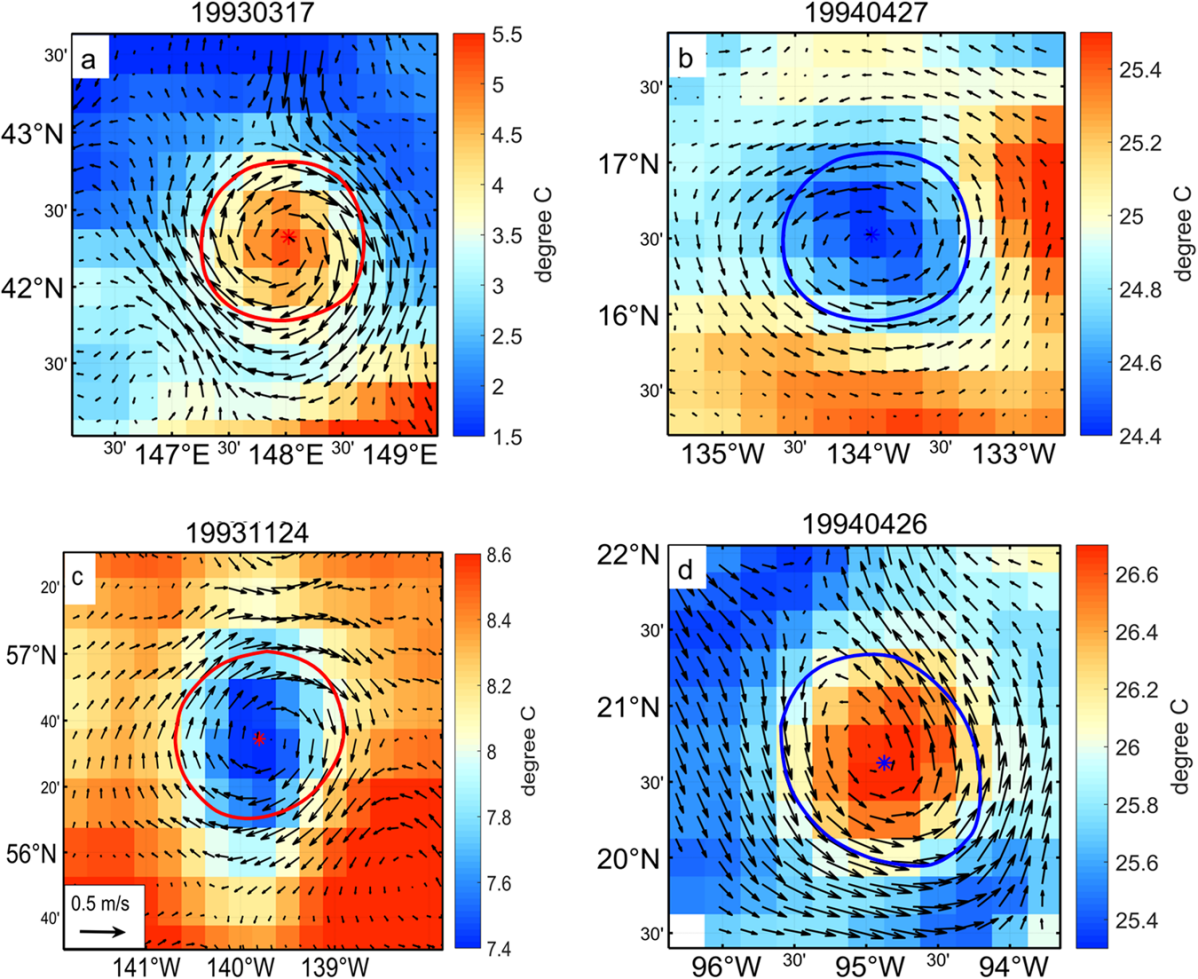
Diagram of normal eddies (top) and abnormal eddies (bottom). The left is listed as anticyclonic eddies and the right as cyclonic eddies. The arrow indicates abnormal sea surface geostrophic, and the color indicates sea surface temperature, “*” indicates the center of the eddy, and the red and blue curve indicates the eddy boundary. The above examples are eddies in the Northern Hemisphere.

Generally and as is commonly understood, cyclonic eddies can induce the upwelling of cooler water from depth, but downwelling occurs for anticyclonic eddies. (Fig. 5a,b). However, the ocean is full of surprises and the appearance of anomalous eddies, i.e., anticyclonic eddies with cold cores (as opposed to warm cores; Fig. 5c) and cyclonic eddies with warm cores (Fig. 5d)^35–37^. From 1993 to 2019, a total of 166,131 abnormal eddies are detected based on every moment, including 82,811 anticyclonic eddies and 83,320 cyclonic eddies.

**Fig. 6:**
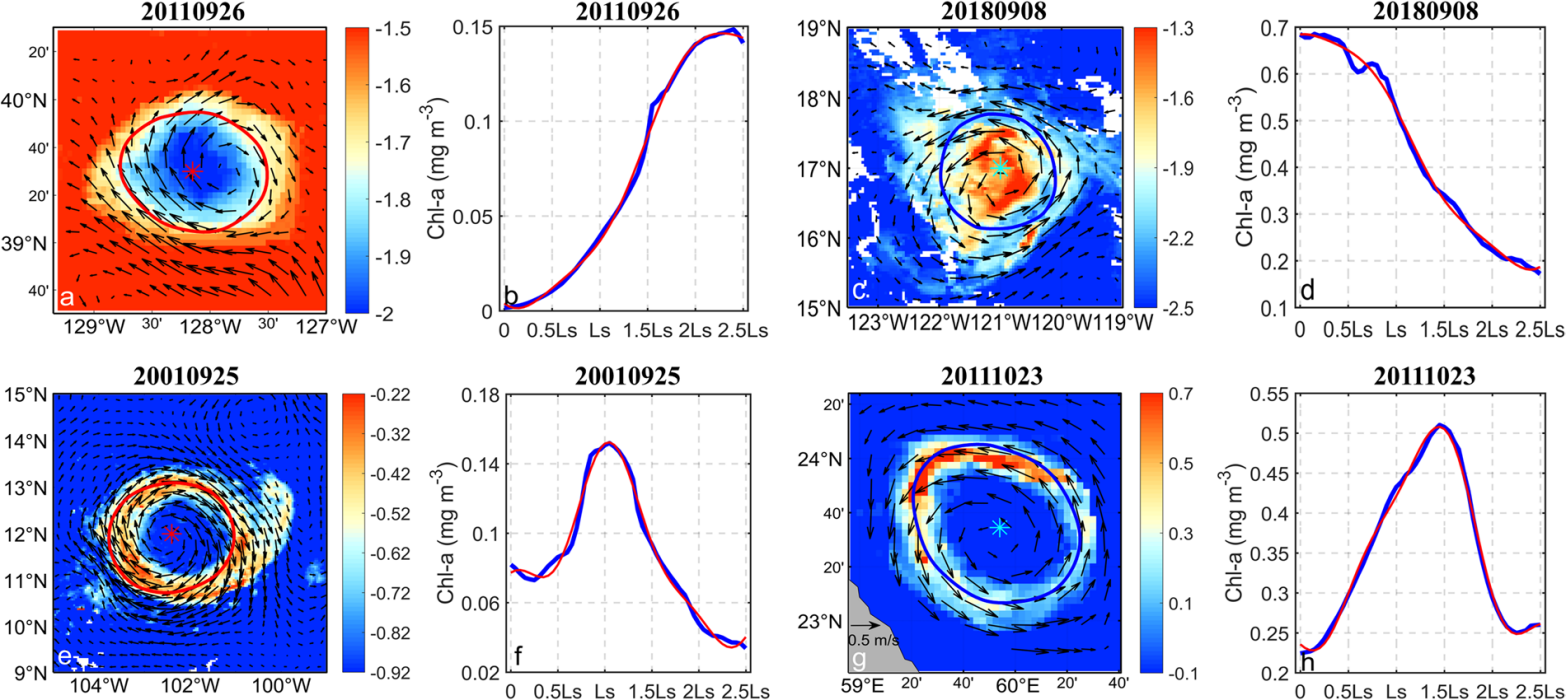
Schematic diagram of abnormal chlorophyll eddies (top) and chlorophyll ring (bottom). (**a**), (**b**), (**e**), (**f**) are anticyclonic eddies, (**c**), (**d**), (**g**), (**h**) are cyclonic eddies. Among them (**a**), (**c**), (**e**), (**g**) are the spatial distribution of chlorophyll on the sea surface around the eddy, the arrow indicates abnormal sea surface geostrophic, and the color indicates the chlorophyll concentration on the sea surface, “*” indicates the center of the eddy, and the red and blue curve indicates the eddy boundary; (**b**), (**d**), (**f**) and (**h**) are the variation trend of sea surface chlorophyll concentration with the eddy radius, blue is the chlorophyll data, red is the fitting data, and Ls is the eddy radius. The above examples are eddy in the northern hemisphere.

As is well understood, mesoscale eddies can transport water masses from one place to another and thus in turn, affect the transport of mass, energy, and momentum. Eddies can also act to stir surrounding water bodies and consequently affect oceanic stratification. Upwelling caused by cyclonic eddies can transport nutrients from depth upward, thereby triggering or sustaining phytoplankton growth rates and chlorophyll content. Downwelling caused by anticyclones can lead to negative anomalies of photosynthetic autotrophs. In this section, the eddy with a life cycle of no less than four weeks is selected for spatiotemporal matching with the SSC data. Differing from pair matching with Argo data, here, the background is defined as data within an area of 2.5 times the eddy center. Each detected eddy is matched with corresponding SSC fields. The distribution of chlorophyll a under eddy action is shown in Fig. 6(a–d). Under the action of eddies of different polarities, the distribution of chlorophyll on the surrounding sea surface is similar to the eddy shape. The chlorophyll distribution is approximately circular around the eddy center. This is different to the distribution of chlorophyll distribution at the outside of the eddy boundary. There, chlorophyll gradually increases (decreases) with the increase of the eddy radius for anticyclonic (cyclonic) eddies. The local extreme value is between the eddy center and a quarter eddy radius. Cyclonic and anticyclonic eddies that could be held responsible for causing SSC anomalies accounted for more than half of the total detected eddies. As shown in Fig. 6(e–h), a small minority of eddies (3.96%) produced what are known as chlorophyll ring structures. The distribution of chlorophyll a on the sea surface under the action of eddies of two polarities is similar, showing a ring structure from the eddy center to the outside, which increases rapidly first and then decreases gradually with the increase of the eddy radius, and shows a local maximum within the eddy radius of 0.5 times to 1.5 times.

**Fig. 7:**
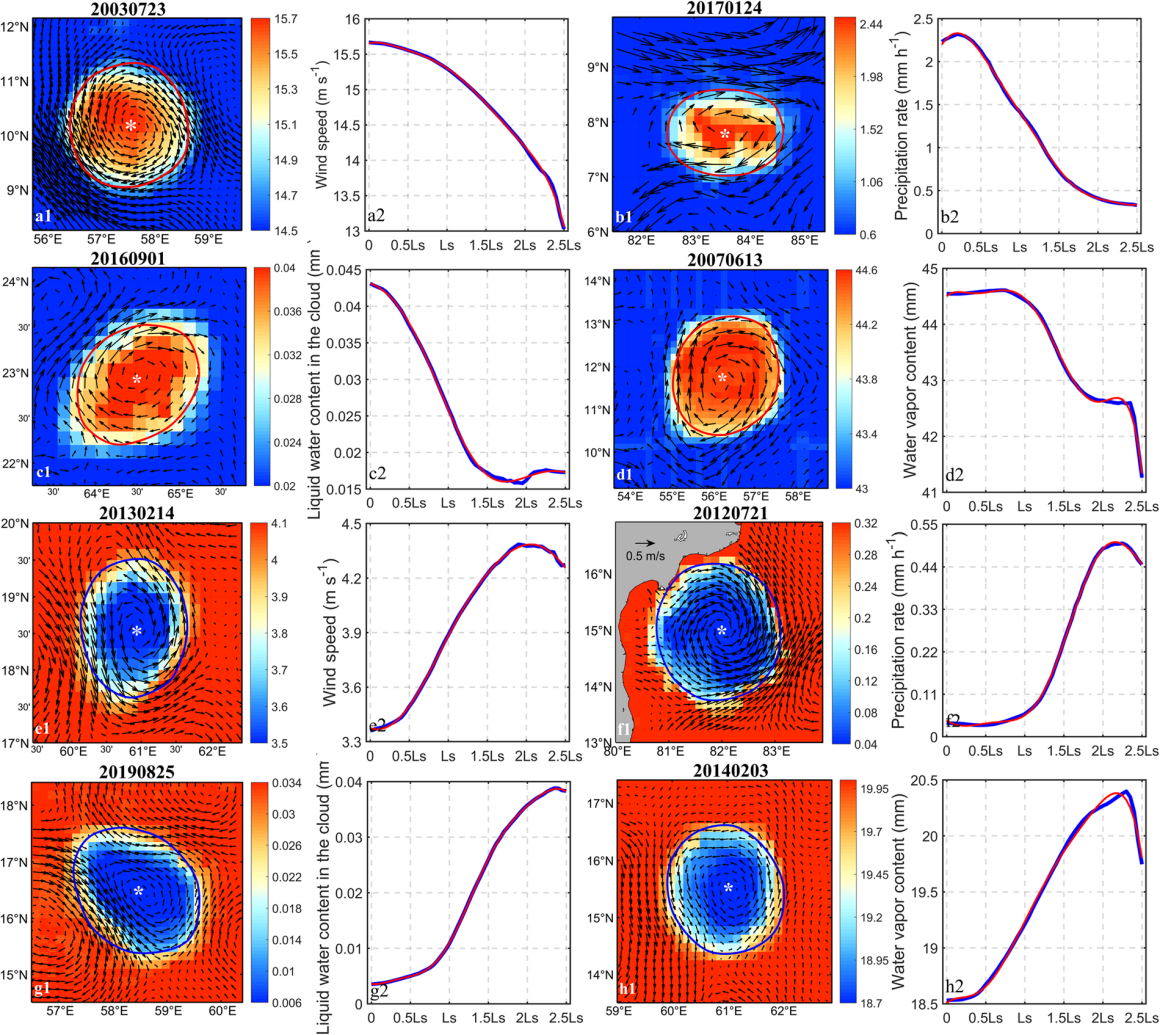
Example diagram of anticyclonic eddies (**a**–**d**) and cyclonic eddies (**e**–**h**) are combined distribution of atmospheric variables. The atmospheric variables are respectively the wind speed on the sea surface (**a**,**e**), the precipitation rate (**b**,**f**), the liquid water content in the cloud (**c**,**g**), and the water vapor content (**d**,**h**). Among them (a1–h1) are the spatial distribution of atmospheric variables around the eddy, the arrow indicates abnormal sea surface geostrophic, “*” indicates the center of the eddy, and the red and blue curve indicates the eddy boundary; (a2–h2) are the variation trend of atmospheric variables with the eddy radius, blue is the atmospheric variables data, red is Fourier fitting data, and Ls is the eddy radius. The above examples are eddy in the northern hemisphere.

Recent literature shows that most mesoscale eddy energy is generated by instabilities in mean flows and air-sea interactions. Mesoscale eddies can and will feed energy and momentum back to the mean flow^53^ which consequently ensures that they can strongly govern global climate change. Here, important atmospheric variables such as sea surface wind speed, liquid water content in clouds, water vapor content and precipitation rate are synthesized with the 4° × 4° region centered on mesoscale eddies. Composite results are shown in Fig. 7. Anticyclonic eddy composites show positive anomalies in atmospheric variables. That is, within eddies, the sea surface wind speed, liquid water content, water vapor content, and precipitation rate all increase. These phenomena are reversed for cyclonic eddies. The maximum values of each variable are in the eddy center and change gradually with the increase of the distance from the center of eddy, which is significantly different from those outside the eddy boundary. This result is consistent with previous research^54^.

## Usage Notes

The near-global ocean mesoscale eddy atmospheric-oceanic-biological interaction observational dataset (GOMED) contains rich information, which can directly provide mesoscale eddies and sea-air generation coupled information data for the study of global mesoscale eddies and related sea-air biological processes. Researchers can determine the required subcategory through their given research area, and then use *eddy_track.nc* to filter the research start time and end time and other parameters to select the eddy serial number that meets the research needs in the database and download the corresponding data.

### Viewing eddy trajectories

According to the eddy tracking file (*eddy_track.nc*), read the eddy observation sequence number and ID, select the eddy time, eddy_type, longitudes and latitudes of eddy center, and longitudes and latitudes of eddy boundary corresponding to the sequence and ID to perform eddy reorganization based on the entire life cycle storage, to get the propagation information of all eddies from generation to disappearance in the whole area. The related program has been uploaded with the eddy dataset, and users can download it by themselves.

### Rebuild eddy sea-air biological information file

Select the file named with a single eddy ID (*ID.nc*) according to the research requirements, read the corresponding eddy parameters (such as ID, eddy_type, time, center, shapes, radius), and obtain the position of the eddy at each moment in the life cycle (the reconstruction of the boundary field is the same as the above). According to the dimensions of the sea-air biological (such as argo_dimension, sst_dimension, ssuv_dimension, amsr_dimension, chl_dimension) matched by the eddy, the background fields of the sea-air biological elements are reconstructed, and the eddy sea-air biological information file is obtained. The related program has been uploaded with the eddy dataset, and users can download it by themselves.

Since the database is provided within several netCDF files, it needs to be read by language software such as MATLAB and Python.

## Data Availability

The mesoscale eddy automatic detection code is available to the public, and can be downloaded from 10.6084/m9.figshare.19802062.v1^55^. It should also be noted that the vector geometry eddy detection algorithm has regional differences, with results deteriorating in coastal zones and near islands, but is still highly accurate for the open ocean. All data processing and calculations in the process of constructing the dataset are completed by MATLAB R2016b (http://www.mathworks.com). Considering user-friendliness, the eddy automatic detection code is currently being converted into a Python format, but due to the huge workload, the code conversion work is still in progress, and the dataset will be uploaded and updated in the future.
